# Chinese Herbal Medicine (*Weijing* Decoction) Combined with Pharmacotherapy for the Treatment of Acute Exacerbations of Chronic Obstructive Pulmonary Disease

**DOI:** 10.1155/2014/257012

**Published:** 2014-08-06

**Authors:** Shaonan Liu, Johannah Shergis, Xiankun Chen, Xuhua Yu, Xinfeng Guo, Anthony Lin Zhang, Chuanjian Lu, Charlie Changli Xue

**Affiliations:** ^1^Guangdong Provincial Hospital of Chinese Medicine, Guangzhou 510120, China; ^2^The 2nd Clinical College of Guangzhou University of Chinese Medicine, Guangzhou 510120, China; ^3^Guangdong Provincial Academy of Chinese Medical Sciences, Guangzhou 510120, China; ^4^Traditional & Complementary Medicine Research Program, Health Innovations Research Institute, School of Health Sciences, RMIT University, Bundoora, Melbourne, VIC 3083, Australia

## Abstract

*Objective*. To evaluate the efficacy and safety of *Weijing* decoction combined with routine pharmacotherapy (RP) for the treatment of acute exacerbations of chronic obstructive pulmonary disease (AECOPD). *Methods*. Randomized controlled trials (RCT) evaluating *Weijing* decoction for AECOPD were included. English, Chinese, and Japanese databases were searched from their respective inceptions to June 2013. The methodological quality was assessed according to the Cochrane Collaboration's risk of bias tool. All data were analyzed and synthesized using RevMan 5.2 software. *Results*. Fifteen (15) studies involving 986 participants were included. Participants were diagnosed with COPD in the acute exacerbation stage. In addition, most of studies reported that they included participants with the Chinese medicine syndrome, phlegm-heat obstructing the Lung. *Weijing* decoction combined with RP improved lung function (forced expiratory volume in one second; FEV1), arterial blood gases (PaO2 and PaCO2), clinical effective rate, and reduced inflammatory biomarkers (TNF-*α* and IL-8) when compared with RP alone. No severe adverse events were reported in these studies. *Conclusions*. *Weijing* decoction appeared to be beneficial for AECOPD and well-tolerated when taken concurrently with RP, such as antibiotics, bronchodilators (oral and inhaled), and mucolytics.

## 1. Introduction

Chronic obstructive pulmonary disease (COPD) is a global disease and is predicted to be the fourth leading cause of death in 2030 [1]. Worldwide COPD affects 9-10% of the adult population [2] and in Asian countries, such as China, the prevalence in people aged over 40 years is 8.2% [3]. COPD is associated with chronic inflammation caused by cigarette smoking and leads to symptoms such as cough, shortness of breath, and increased sputum production [4]. One factor that is of widespread concern is the occurrence of acute exacerbations of COPD (AECOPD). AECOPD is diagnosed clinically when patients present with worsening of dyspnoea, cough and/or sputum that is greater than day-to-day variations. Acute exacerbations may require hospitalization or change in medications and can lead to lung function decline and reduced quality of life [4].

Exacerbations of COPD are commonly treated with drugs such as bronchodilators, corticosteroids, and antibiotics and in more severe cases may require oxygen therapy and ventilator support. These therapies are beneficial; however they can lead to some significant side effects such as headache, insomnia, nausea, and pneumonia [4]. Therefore, there is still a need to improve the management of AEOCPD.

Chinese herbal medicine (CHM) showed potential beneficial effect for AECOPD in a recent systematic review; lung function, arterial blood gases, and clinical effective rate were improved by a Chinese herbal formula* Tan re qing* [5]. Despite* Tan re qing's* positive effects this formula is not commonly used outside of China and is given intravenously, which is not the traditional delivery method of CHM. A universally used herbal formula for treating AECOPD is* Weijing* decoction [6]. It contains four herbs* Weijing* (*Coulis phragmitis*),* tao ren* (*Semen persicae*),* yi yi ren* (*Semen coicis*), and* dong gua ren* (*Semen benincasae*) and has been used as a traditional herbal combination for thousands of years [7]. It treats the Chinese medicine syndrome, “phlegm and heat obstructing the Lung,” which is one of the most common syndromes associated with AECOPD [8].

Clinical trials have demonstrated that* Weijing* decoction combined with pharmacotherapy can improve symptoms, lung function, and arterial blood gases during AECOPD [9, 10]. In addition, the herbs included in* Weijing* decoction improved immunity and reduced bacterial load* in vitro* and* in vivo* animal studies [11–13]. Comprehensive analysis of* Weijing* decoction is not available. Therefore, the systematic review and meta-analysis evaluate the efficacy and safety of* Weijing* decoction combined with RP for treating AECOPD.

## 2. Methods

### 2.1. Study Selection

Included studies were randomized controlled trials (RCT) investigating* Weijing* decoction for the treatment of acute exacerbation of COPD (AECOPD). Intervention was oral* Weijing* decoction combined with routine pharmacotherapy (RP) versus the same routine pharmacotherapy alone in the control group. Included participants were diagnosed with AECOPD according to the Global Initiative for Chronic Obstructive Lung Disease [4]; British Thoracic Society; American Thoracic Society; European Respiratory Society; British Medical Research Council; or Chinese COPD guidelines [14].

Several broad outcome measures were selected to provide critical data on measuring different aspects of AECOPD. The outcome measures include lung function; dyspnoea; health related quality of life, emergency department or hospital admissions; length of hospital stay; arterial blood gases—partial pressure of oxygen (PaO2) and carbon dioxide (PaCO2); biomarkers, tumour necrosis factor alpha (TNF-*α*) and interleukin (IL)-8; or clinical effective rate. Clinical effective rate was defined as an improvement in symptoms, such as cough, sputum production, and dyspnoea. The improvement was judged by a clinician and based on COPD guidelines [15].

Studies were excluded if they combined* Weijing* decoction with other Chinese medicine therapies, such as acupuncture and/or Chinese herbal medicine administered as an injection, or the comparator RP was not a medication recommended by COPD guidelines.

### 2.2. Search Strategy

The search was conducted in five English (PubMed, EMBASE, the Cochrane Central Register of Controlled Trials (CENTRAL), CINAHL, and AMED) and four Chinese (Chinese Biomedical Database (CBM), Chinese National Knowledge Infrastructure (CNKI), Wanfang database, and Chongqing VIP information (CQVIP)) databases. The search time frame ranged from the databases' inception until 9 June 2013. No restrictions were applied. To ensure the largest sample of herbal formulae was included,* Weijing* decoction was not specifically searched. The search terms were selected to identify any study that used herbal medicine which may or may not have included* Weijing* decoction. In addition, the supplementary search was conducted in two Korean (Research Information Service System (RISS), National Library of Korea) and two Japanese (J-STAGE, Ichushi WEB 4.0) databases with specified* Weijing* decoction term. The search included terms for chronic obstructive pulmonary disease, traditional Chinese medicine, and randomized controlled trial. The full list of search terms is in the Appendix.

### 2.3. Data Selection and Extraction

Two independent researchers (Shaonan Liu and Xuhua Yu) screened the studies according to the eligibility criteria and disagreement was resolved by a third researcher (Xiankun Chen).

Recorded information on study characteristics included first author, publication year, location, setting, study design, population characteristics, sample size,* Weijing* decoction ingredients, dose, administration, study duration, outcome measures, and adverse events. Study authors were contacted for missing and incomplete data.

### 2.4. Risk of Bias Assessment

The methodological quality was assessed by three independent researches (Shaonan Liu, Xiankun Chen, and Xuhua Yu) according to the Cochrane Collaboration's risk of bias tool [16]. Seven sources of bias were assessed including sequence generation, allocation concealment, blinding of participants and personnel, blinding of outcome assessors, incomplete outcome data, selective reporting, and other bias. Other bias included funding source, conflicts of interests, and baseline imbalance. The author was contacted through telephone if the methodological information was not very clear (Xuhua Yu). Any discrepancies were resolved by another reviewer (Xinfeng Guo).

### 2.5. Data Analysis

All data were analysed and synthesized using RevMan 5.2 software. Dichotomous data were calculated and presented as risk ratio (RR) with 95% confidence intervals (95% CI), and continuous data were reported as mean difference (MD) and 95% CI, and standardized mean difference (SMD) was used when studies reported different scales of the same outcome measure. Statistical heterogeneity was evaluated using Chi-square test and *I* ^2^ test. A fixed-effect model was used if *I* ^2^ was less than 50%; otherwise a random-effects model was applied. Sensitivity analysis was performed and included studies at low risk of bias for random sequence generation and subgroup analysis was performed based on the different control treatments. Publication bias will be assessed using a funnel plot if nine or more studies were included in the meta-analysis and Begg's rank correlation test was used if symmetry was unclear.

## 3. Results

### 3.1. Description of Studies

**Figure 1:**
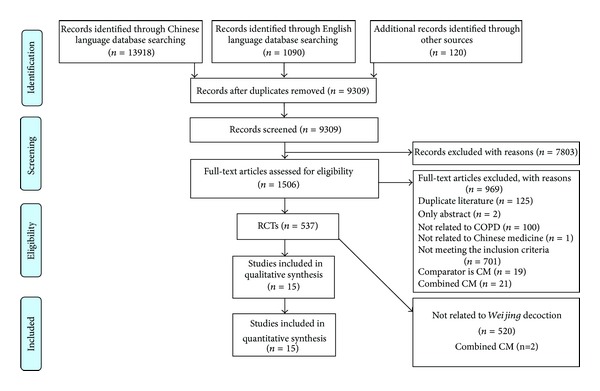
Flow chart of the study selection process.

The search identified 15,128 publications. After duplicates were removed, 9,309 studies were screened and 1,506 full texts were reviewed. After full-text review 537 RCTs using Chinese herbal medicine were reviewed. Only 15 of these studies used* Weijing* decoction combined with RP and were included in this review [9, 10, 17–29].  Figure 1 presents the details. All studies were identified from the Chinese literature and were conducted in China. The 15 studies included 986 participants with intervention group including 508 cases and 478 cases for control group and sample sizes ranging from 40 to 100. The treatment duration ranged from 7 to 15 days and none of the studies included a follow-up period. Severity of COPD was reported in seven studies [9, 10, 21, 22, 24, 26, 27] and participants were at all stages of COPD from mild to very severe.

All studies used one packet of* Weijing* decoction (with modifications) taken twice a day. Four studies combined* Weijing *decoction with another oral herbal formula to treat other symptoms associated with COPD such as those caused by* Lung Qi deficiency*. Formulae included* Ma xing shi gan* decoction (2 studies),* Er chen,* and* Liu jun zi *decoction (1 study each) [18, 23, 25, 26]. Comparator types were grouped as specified routine pharmacotherapy (SRP) (e.g., levofloxacin, salbutamol, ipratropium, theophylline, and ambroxol hydrochloride) or unspecified routine pharmacotherapy (URP) (e.g., bronchodilators, antibiotics, and mucolytics). The intervention group of all trials received the same pharmacotherapy as the control groups. Seven studies specified the pharmacotherapy [10, 17, 19, 20, 24, 25, 27] and eight did not [9, 18, 21–23, 26, 28, 29]. For the studies that specified pharmacotherapy, they all used a combination of antibiotics, bronchodilators (oral and/or inhaled), mucolytics, and oxygen therapy. Chinese medicine syndromes were reported in 12 studies: phlegm-heat obstructing the Lung (11 studies), phlegm-heat obstructing the Lung combined with Lung and Spleen Qi deficiency (1 study) [23] and three studies did not specify the Chinese medicine syndrome [18, 25, 28]. Study characteristics are presented in Table 1.

### 3.2. Assessment of Risk of Bias

**Figure 2:**
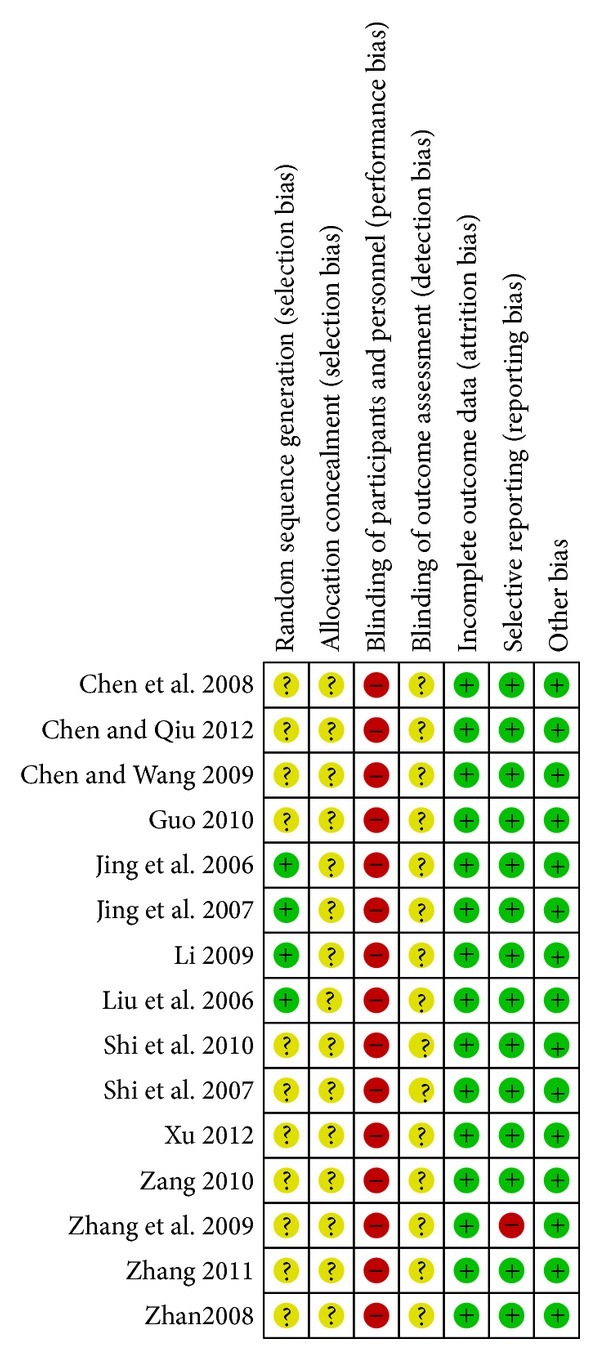
Assessment of risk of bias.

All studies were described as randomized. However only four reported the details of random sequence generation using appropriate methods such as a random number table [9, 10, 21, 22]. The authors of 11 studies were contacted, with no response, so these studies were assessed with unclear risk of bias. Allocation concealment was not described in any of the studies and blinding of participants and personnel was not performed. Blinding of outcome assessors was also not described in any of the studies and they were judged to be at unclear risk of bias. None of the studies had drop-outs and therefore incomplete outcome data was judged at low risk of bias. All trials reported outcomes consistent with the methods section except one which reported more outcomes and was therefore judged at high risk of bias. Other bias was judged at low risk of bias in all the studies (see Figure 2).

### 3.3. Publication Bias

Studies reporting FEV1% and effective rate were evaluated for publication bias. Visual inspection was unclear (Figures 8 and 9); therefore we performed Begg's rank correlation test. For FEV1% there was no publication bias (*Z* = 1.15, *P* = 0.251). However, for effective rate, there was statistically significant publication bias (*Z* = 2.96, *P* = 0.003).

### 3.4. Outcome Measures

#### 3.4.1. Lung Function

**Figure 3:**
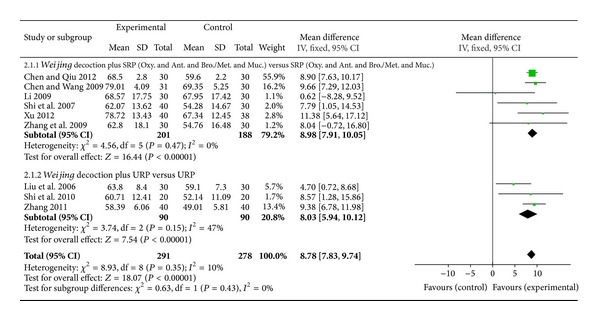
Forest plot of* Weijing* decoction plus RP versus RP for effect on FEV1%: 2.1.1* Weijing* decoction plus SRP; 2.1.2* Weijing* decoction plus URP. RP: routine pharmacotherapy, SRP: specified routine pharmacotherapy, URP: unspecified routine pharmacotherapy, Oxy.: oxygen therapy, Ant.: antibiotic, Bro.: bronchodilators, Met.: methylxanthines, and Muc.: mucolytics.

**Figure 4:**
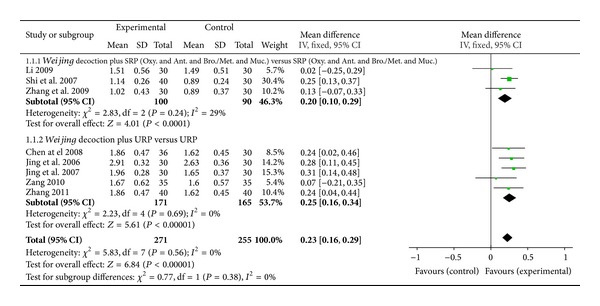
Forest plot of* Weijing* decoction plus RP versus RP for effect on FEV1: 1.1.1* Weijing* decoction plus SRP; 1.1.2* Weijing* decoction plus URP. RP: routine pharmacotherapy, SRP: specified routine pharmacotherapy, URP: unspecified routine pharmacotherapy, Oxy.: oxygen therapy, Ant.: antibiotic, Bro.: bronchodilators, Met.: methylxanthines, and Muc.: mucolytics.

For lung function FEV1 percentage predicted (FEV1%), nine studies were included.* Weijing* decoction in combination with RP improved FEV1% compared with the same RP (MD 8.78%, 95% CI 7.83 to 9.74, *I* ^2^ = 10%). When* Weijing *decoction was combined with antibiotics, bronchodilators (oral and/or inhaled), mucolytics, and oxygen therapy, FEV1% improved compared with the control group (6 studies, MD 8.98%, 95% CI 7.91 to 10.05, *I* ^2^ = 0%) (Figure 3). In a meta-analysis for FEV1 litres (Figure 4), there was significant improvement in favour of intervention (8 studies, MD 0.23L, 95% CI 0.16 to 0.29, *I* ^2^ = 0%). After subgrouping analysis by specific drugs (combination of antibiotics, bronchodilators, and mucolytics) FEV1 litres also improved (3 studies, MD 0.20L, 95% CI 0.10 to 0.29, *I* ^2^ = 29%) (Figure 4).

Sensitivity analysis after removal of studies at high or unclear risk of bias for random sequence generation showed positive effects of* Weijing* plus RP compared with RP alone; FEV1 litres (2 studies, MD 0.25L, 95% CI 0.14 to 0.36 *I* ^2^ = 41%); and FEV1% (2 studies, MD 4.02, 95% CI 0.38 to 7.65, *I* ^2^ = 0%) (Table 2).

#### 3.4.2. Arterial Blood Gas Analysis

**Figure 5:**
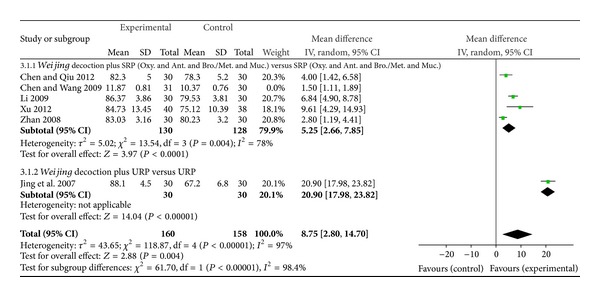
Forest plot of* Weijing* decoction plus RP versus RP for effect on PaO2: 3.1.1* Weijing* decoction plus SRP; 3.1.2* Weijing* decoction plus URP. RP: routine pharmacotherapy, SRP: specified routine pharmacotherapy, URP: unspecified routine pharmacotherapy, Oxy.: oxygen therapy, Ant.: antibiotic, Bro.: bronchodilators, Met.: methylxanthines, and Muc.: mucolytics.

**Figure 6:**
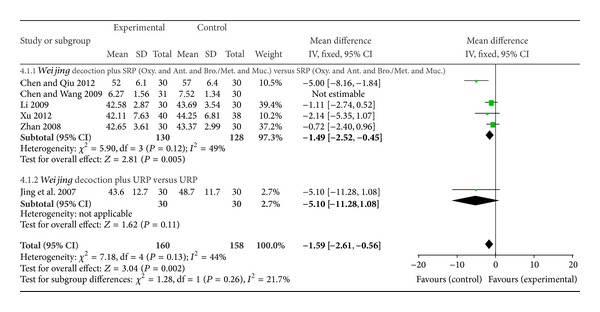
Forest plot of* Weijing* decoction plus RP versus RP for effect on PaCO2: 4.1.1* Weijing* decoction plus SRP; 4.1.2* Weijing* decoction plus URP. RP: routine pharmacotherapy, SRP: specified routine pharmacotherapy, URP: unspecified routine pharmacotherapy, Oxy.: oxygen therapy, Ant.: antibiotic, Bro.: bronchodilators, Met.: methylxanthines, and Muc.: mucolytics.

Arterial blood gases were reported as millimetres mercury (mmHg) except for one study that used kilopascals (Kpa) [19]. It is difficult to interpret the results in clinical practice when SMD was used to do the pooled-analysis; therefore, the one study that used Kpa was not merged for analysis. From five studies analysed PaO2 showed a significant improvement (MD 8.75 mmHg, 95% CI 2.80 to 14.70); however studies were heterogeneous *I* ^2^ = 97%. Subgroup analysis by specific pharmacotherapy also showed positive effects (4 studies, MD 5.25 mmHg, 95% CI 2.66 to 7.85, *I* ^2^ = 78%) (Figure 5). Reduction in PaCO2 was also shown (5 studies, MD −1.59 mmHg, 95% CI −2.61 to −0.56, *I* ^2^ = 44%), after subgroup analysis (4 studies MD −1.49 mmHg, 95% CI −2.52 to −0.45, *I* ^2^ = 49%) (Figure 6). Sensitivity analysis showed similar effects with the larger pool for PaO2 (2 studies, MD 13.83, 95% CI 0.05 to 27.60, *I* ^2^ = 98%) and PaCO2 (2 studies, MD −1.37 mmHg, 95% CI −2.95 to 0.21, *I* ^2^ = 33%) (Table 2).

#### 3.4.3. Clinical Effective Rate

**Figure 7:**
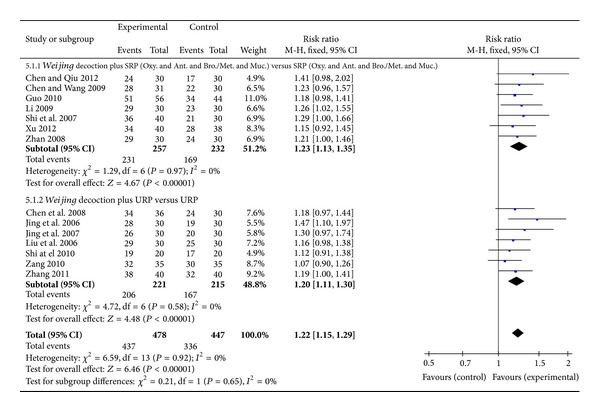
Forest plot of* Weijing* decoction plus RP versus RP for effect on effective rate: 5.1.1* Weijing* decoction plus SRP; 5.1.2* Weijing* decoction plus URP. RP: routine pharmacotherapy, SRP: specified routine pharmacotherapy, URP: unspecified routine pharmacotherapy, Oxy.: oxygen therapy, Ant.: antibiotic, Bro.: bronchodilators, Met.: methylxanthines, and Muc.: mucolytics.

**Figure 8:**
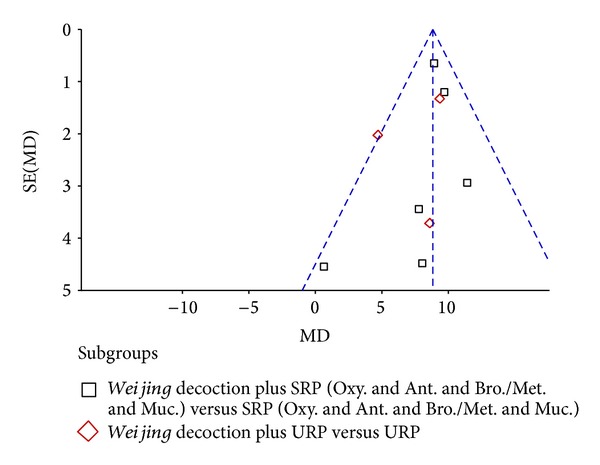
Funnel plot of publication bias using FEV1%.

**Figure 9:**
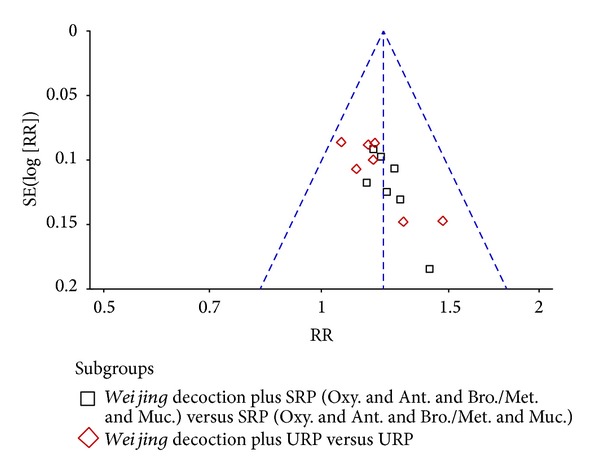
Funnel plot of publication bias using effective rate.

Effective rate was assessed and based on clinician's judgment of symptom improvement, mostly using the “Guiding Principles of Clinical Research on New Drugs of Traditional Chinese Medicine” [15]. This guideline described effectiveness of an intervention by its ability to reduce sputum production and cough, and so forth. Fourteen studies were included for effective rate outcome. Results favoured the* Weijing *decoction group (RR 1.22, 95% CI 1.15 to 1.29, *I* ^2^ = 0%). In seven studies,* Weijing* decoction combined with antibiotics, bronchodilators (oral and/or inhaled), mucolytics, and oxygen therapy compared with pharmacotherapy alone also showed a significant effect (RR 1.23, 95% CI 1.13 to 1.35, *I* ^2^ = 0%) (Figure 7).

Four studies were included in the sensitivity analysis. The result was similar with the larger pool of studies (RR 1.29, 95% CI 1.14 to 1.45, *I* ^2^ = 0%) (Table 2).

#### 3.4.4. Biomarkers

Four studies were included in the analysis of serum TNF-*α*.* Weijing* decoction plus RP reduced TNF-*α* (SMD −3.47, 95% CI −5.39 to −1.55, *I* ^2^ = 97%) compared with RP alone. It also reduced IL-8 in five studies (SMD −0.84, 95% CI −1.11 to −0.57, *I* ^2^ = 98%).

#### 3.4.5. Other Outcomes

Four predefined outcomes were not reported in any of the included studies. These outcomes were dyspnoea, health related quality of life, emergency department or hospital admissions, and length of hospital stay.

### 3.5. Adverse Events

Seven out of the 15 studies reported adverse events [9, 19, 21, 25, 27–29]. Six studies reported that no adverse events occurred [9, 19, 21, 25, 27, 28] and one study reported 10 adverse events in the intervention group and 8 events in the control group [29]. Adverse events included mild abnormal liver function test results (intervention group: 3 cases, control: 2 cases), mild abnormal kidney function test results (intervention group: 1 case, control: 2 cases), and gastrointestinal upset (intervention group: 6 cases, control: 4 cases). No causality assessment was conducted for these adverse events. No severe adverse events were reported.

## 4. Discussion

This review based on published RCT revealed that* Weijing* decoction in conjunction with RP were more effective in improving outcomes for AECOPD including lung function, arterial blood gases, and clinical effective rate, when compared with RP alone.

In terms of lung function,* Weijing* decoction plus RP improved FEV1 litres by 0.23 litres and FEV1% increased by 8.78%. These results although not clinically significant for stable COPD may be clinically significant during AECOPD because even small increases in lung function may shorten recovery time [4]. Measurement of blood gases was also improved after Weijing plus RP. Blood gases are useful for AECOPD evaluation because they can predict survival rates in hospitalized patients and define respiratory failure and hypoxaemia/hypercapnia [30]. Participants taking* Weijing* decoction showed increased PaO2 and PaCO2 indicating improvement in health status and reduced likelihood of respiratory failure.

Effective rate assessed by clinician's judgment of symptom improvement is a common outcome used in Chinese medicine clinical trials. In this review effective rate was evaluated in all but one study and pooled data showed a significant difference between the groups, in favour of* Weijing* decoction. However for this outcome publication bias was detected and results need careful interpretation. The results were statistically significant but small. However these small increases may be clinically significant during AECOPD.

*Weijing* decoction plus RP compared with RP alone reduced TNF-*α* and IL-8 in blood serum/plasma. Markers of inflammation, such as cytokines, TNF-*α*, and IL-8, are elevated in AECOPD [31, 32] and studies indicate that reducing inflammatory markers can shorten recovery time and reduce recurrence of AECOPD [33]. Despite this, there is no consensus on the use of inflammatory biomarkers for predicting COPD progression or response to therapy. They are considered to be an important outcome that would allow more precise diagnosis and once fully established should be considered as outcomes for COPD clinical trials [4].

*Weijing* decoction was safe for AECOPD and well-tolerated in combination with RP. Adverse events reported in the trials included abnormal liver and kidney function tests and gastrointestinal upset. However these events were considered to be mild and there was no difference between groups.

Findings from this review are comparable with previous systematic reviews that evaluated Chinese herbal medicine combined with RP for AECOPD [5, 34]. These reviews also evaluated lung function, arterial blood gases, and clinical effective rate and the study participants were diagnosed with the Chinese medicine syndrome, phlegm-heat obstructing the Lung. One review used* Tan re qing* injection and included 14 trials involving 954 participants.* Tan re qing* injection combined with pharmacotherapy improved lung function, clinical efficacy, and arterial blood gas and shortened the length of hospital stay compared with pharmacotherapy [5]. In the other review 16 studies used* Da cheng qi* decoction combined with pharmacotherapy and all outcomes were improved (lung function, clinical efficacy, and arterial blood gas) [34].

Although previous reviews have been published, this review evaluated a commonly used and recommended oral herbal formula for AECOPD.* Tan re qing* injection is also commonly used; however it is given by injection and not widely used outside of China. The other review used* Da chenq qi* decoction [34]. This herbal formula is not used or recommended to treat AECOPD, unless the patient has accompanied digestive tract symptoms such as abdominal distension and constipation.

Several limitations should be considered when interpreting this study. There were methodological shortfalls in the included studies. Only a small number of trials included information on randomization and blinding of participants and personnel was not performed in any of the trials. The effects shown by the result of sensitivity analysis were similar to the total analysis; however, the methodological shortfalls may cause potential risk of bias and influence the reliability of the conclusion. Therefore, CONSORT statement was also recommended for RCTs. Sample sizes were also small and a calculation of sample size was not performed in included studies. The predefined outcomes dyspnoea, health related quality of life, emergency department or hospital admissions, and length of hospital stay were not reported in any of the included studies. These outcomes especially dyspnoea and health related quality of life are useful when assessing and monitoring outcomes in patients with AECOPD and can be good predictors of future mortality risk [35, 36]. Analysis of these outcomes would have improved understanding of the effects of* Weijing* decoction and add to a more comprehensive recommendation for clinical practice.

## 5. Conclusions

Despite methodological limitations of the included studies,* Weijing* decoction combined with RP appears to be effective for the treatment of AECOPD in terms of improving lung function, arterial blood gases, and clinical effective rate and reducing inflammatory markers. Future studies should include proper randomization methods and blinding of participants and personnel as well as recording and reporting adverse events. In terms of Chinese medicine practice,* Weijing *decoction may provide benefit to individuals with AECOPD. Very few side effects were reported and* Weijing* decoction appears safe for AECOPD patients in combination with antibiotics, bronchodilators (oral and/or inhaled), and mucolytics.

## Figures and Tables

**Table 1:** Characteristics of included studies.

First author, publication year, country, setting	Treatment duration	Severity; duration of condition	Number of participants randomised/assessed	Age (mean (SD) or range); gender (M/F)	Intervention∗	Control (pharmacotherapy)
Chen et al., 2008 [18], China, inpatients	2 w	NS; I: 9.8 (5.0) y C: 9.5 (4.6) y	I: 36/36 C: 30/30	I: 65.7 (8.3); 24/12 C: 64.8 (8.0); 18/12	*Weijing* decoction and Er chen decoction	Routine care (oxygen therapy, bronchodilators, antibiotics, mucolytics plus nutritional therapy) not specified

Jing et al., 2007 [9], China, inpatients	1 w	Mild-moderate; I: 17.2 (3.5) y C: 13.5 (3.7) y	I: 30/30 C: 30/30	I: 66.3 (5.4); 22/8 C: 67.2 (4.4); 20/10	Qian jin *Weijing* decoction	Routine care (bronchodilators, antibiotics, mucolytics plus nutritional therapy) not specified

Liu et al., 2006 [22], China, NS	10 d	I: mild-very severe; 16.86 (10.97) y C: mild-very severe; 17.20 (11.25) y	I: 30/30 C: 30/30	I: 69.17 (7.53); 21/9 C: 69.05 (7.83); 22/8	Jia wei qian jin *Weijing* decoction	Routine care (oxygen, antibiotics plus nutritional therapy) not specified

Chen and Qiu, 2012 [17], China, inpatients	2 w	I: NS; 16.79 (10.53) y/ C: NS; 17.20 (11.25) y	I: 30/30 C: 30/30	I: 72.07 (8.39); 20/10 C: 71.05 (7.93); 22/8	Qian jin *Weijing* decoction	Oxygen therapy, Methylxanthines (Doxofylline 0.3 g iv qd); Ventolin 2 spray inhaled, Ipratropium 10 mL qd inhaled, Levofloxacin, 0.4 g, IV, qd; ambroxol hydrochloride, 30 mg, IV, tid;

Shi et al., 2007 [24], China, inpatients	2 w	I: mild: 4, moderate: 29, severe: 7, 16.34 (9.53) y/ C: mild: 2, moderate: 19, severe: 9; 17.17 (10.22) y	I: 40/40 C: 30/30	I: 61.4 (6.8); 27/13 C: 59.5 (7.2); 18/12	*Weijing* xuan bi decoction	Oxygen therapy, antibiotics (Cefmetazole 1 g iv bid) plus methylxanthines (Aminophylline 0.1 g, bid) plus mucolytic (Mucosolvan 30 mg bid)

Zhang et al., 2009 [28], China, inpatients	10 d	NS	I: 30/30 C: 30/30	I: 65.21 (6.02); 25/5 C: 65.30 (6.13); 23/7	Qian jin *Weijing* decoction	Oxygen therapy, antibiotics plus methylxanthines (Theophylline 0.2 g, bid) plus mucolytic (Mucosolvan 30 mg tid)

Chen and Wang, 2009 [19], China, inpatients	15 d	NS	I: 31/31 C: 31/31	I: 62.5 (NS); 23/8 C: 61.7 (NS); 22/9	*Weijing* decoction	Oxygen therapy, Cefalexin, 3 g, qd-bid; ipratropium, 2 mL, inhaled, tid; salbutamol, 1 mL, inhaled, tid; theophylline, 0.2 g, bid; Mucosolvan, 30 mg, tid

Li, 2009 [10], China, inpatients	10 d	I: mild: 5, moderate: 25; 9.94 (3.62) y/ C: mild: 6, moderate: 24; 8.98 (3.41) y	I: 30/30 C: 30/30	I: 63.87 (8.64); 19/11 C: 63.62 (7.23); 18/12	Qian jin *Weijing* decoction	oxygen therapy, Levofloxacin, 0.3 g, IV, qd; salbutamol, 200 ug, inhaled tid, ipratropium, 20 ug, inhaled, tid, theophylline, 0.2 g, PO, bid; ambroxol hydrochloride, 30 mL, tid

Shi et al., 2010 [23], China, NS	10 d	NS	I: 20/20 C: 20/20	Total: 52–83; 31/9	*Weijing* decoction and liu jun zi decoction	Routine care (oxygen therapy, bronchodilators, antibiotics, mucolytics, and others) not specified

Jing et al., 2006 [21], China, inpatients	10 d	mild-moderate; I: 13.2 (3.7) y C: 12.8 (3.9) y	I: 30/30 C: 30/30	I: 64.7 (5.2); 21/9 C: 64.2 (4.9); 18/12	Qian jin *Weijing* decoction jia jian	Routine care (bronchodilators, antibiotics, mucolytics plus nutritional therapy) not specified

Xu, 2012 [25], China, out/inpatients	2 w	I: NS; 12.24 (3.79) y/ C: NS; 15.29 (5.34) y	I: 40/40 C: 38/38	I: 65.82 (11.73); 23/17 C: 64.15 (13.84); 21/17	Qian jin *Weijing* decoction and ma xing shi gan decoction	Oxygen therapy, antibiotics plus methylxanthines (Aminophylline) plus mucolytic (Mucosolvan)

Zang, 2010 [26], China, inpatients	2 w	Mild-severe; I: 11.6 (NS) d C: 10.4 (NS) d	I: 35/35 C: 35/35	I: 62.3 (NS); 23/12 C: 61.7 (NS); 24/11	Qian jin *Weijing* decoction and ma xing shi gan decoction	Routine care (bronchodilators, antibiotics, mucolytics plus nutritional therapy) not specified

Zhang, 2011 [29], China, NS	10 d	NS	I: 40/40 C: 40/40	Total: 67.7 (7.1); 59/21	Qian jin *Weijing* decoction (modified)	Routine care (oxygen therapy, bronchodilators, antibiotics, mucolytics) not specified

Zhan, 2008 [27], China, out/inpatients	10 d	I: mild: 5, moderate: 13, severe: 12; 15.46 (8.37) y/ C: mild: 5, moderate: 15, severe: 10; 16.01 (2.68) y	I: 30/30 C: 30/30	I: 65.7 (8.54); 25/5 C: 64.9 (9.61); 23/7	*Weijing* decoction	Oxygen therapy, Levofloxacin, 0.3 g, IV, qd methylxanthines (Aminophylline 0.1 g, bid) plus mucolytic (ambroxol, 30 mg, tid)

Guo, 2010 [20], China, inpatients	15 d	I: NS; 8.9 y C: NS; 9.3 y	I: 56/56 C: 44/44	I: 63.5; 30/26 C: 68.5; 21/23	*Weijing* decoction	Oxygen therapy, antibiotics plus methylxanthines (Theophylline 0.2 g, bid) plus mucolytic (Mucosolvan 30 mg tid)

I: intervention; C: control; IV: intravenous; NS: not specified; d: day; w: week; y: year.

∗In all studies the same pharmacotherapy was used in the intervention group as in the control group.

**Table 2:** Sensitivity analysis.

Outcome	Number of studies	Effect estimate MD/RR (95% CI)
FEV1	3 (Li 2009 [10], Jing et al., 2006 [21], Jing et al., 2007 [9])	MD 0.25 (0.14, 0.36)
FEV1%	2 (Li 2009 [10], Liu et al., 2006 [22])	MD 4.02 (0.38, 7.65)
PaO2	2 (Li 2009 [10], Jing et al., 2007 [9])	MD 13.83 (0.05, 27.60)
PaCO2	2 (Li 2009 [10], Jing et al., 2007 [9])	MD −5.10 (−11.28, 1.08)
Effective rate	4 (Li 2009 [10], Jing et al., 2006 [21], Jing et al., 2007 [9], Liu et al., 2006 [22])	RR 1.29 (1.14, 1.45)

Sensitivity analysis removed studies with unclear or high risk of bias for sequence generation.

## Appendix

### Search Strategies

*English Databases*. #1: Pulmonary Disease, Chronic Obstructive OR Bronchitis, Chronic OR Pulmonary Emphysema OR Emphysema OR COPD OR Chronic Obstructive Pulmonary OR COAD OR AECB OR COBD OR Chronic Obstructive Airway OR Chronic Obstructive Lung OR Chronic obstructive bronchopulmonary OR Chronic obstructive respiratory OR Chronic Airflow Obstruction OR Chronic Airflow Obstructive OR Chronic bronchitis OR Pulmonary emphysema OR Lung emphysema OR Chronic Airflow limitation.

#2: Traditional Chinese Medicine OR Chinese Traditional Medicine OR Chinese Herbal Drugs OR Chinese Drugs, Plant OR Medicine, Traditional OR Ethnopharmacology OR Ethnomedicine OR Ethnobotany OR Medicine, Kampo OR Kanpo OR TCM OR OR Medicine, Ayurvedic OR Phytotherapy OR Herbology OR Plants, Medicinal OR Plant Preparation OR Plant Extract OR Plants, Medicine OR Materia Medica OR Single Prescription OR Herbs OR Chinese Medicine Herb OR Herbal Medicine.

#3: Randomized controlled trial or controlled clinical trial or randomized or placebo or drug therapy or randomly or trial or groups.

#4: #1 AND #2 AND #3.

*Chinese Databases (Search by Using Simplified Chinese Character)*. #1: Man Xing Zu Sai Xing Fei Bing (Chronic obstructive pulmonary disease) OR Man Zu Fei (Chronic obstructive pulmonary disease) OR COPD OR Fei Qi Zhong (Obstructive pulmonary emphysema) OR Man Xing Zhi Qi Guan Yan (Chronic bronchitis) OR Zu Sai Xing Fei Bing (Chronic obstructive pulmonary disease) OR Zu Sai Xing Fei Ji Bing (Chronic obstructive pulmonary disease).

#2: Zhong Yi (Traditional Chinese Medicine) OR Zhong Xi Yi (Integrative medicine) OR Zhong Yi Liao Fa (Chinese Medicine Therapeutics) OR Bian Zheng Lun Zhi (syndrome differentiation and treatment) OR Bian Zheng (syndrome differentiation) OR Han Fang (Kampo) OR Zu Guo Yi Xue (Chinese Medicine) OR Chuan Tong Yi Xue (traditional medicine) OR Chuan Tong Zhi Liao (traditional treatment) OR Ti Dai Yi Xue (Complementary disease) OR Ti Dai Zhi Liao (complementary treatment) OR Zhong Guo Chuan Tong Yi Xue (traditional Chinese medicine) OR Min Zu Yi Yao (Ethnomedicine) OR Cao Yao (herbal medicine) OR Zhong Cao Yao (Chinese herb medicine) OR Zhong Yao Liao Fa (Chinese herb medicine therapeutics) OR Zhong Xi Yao (Chinese and western medicine) OR Zhong Cheng Yao (Chinese patent medicine).

#3: Lin Chuang Guan Cha (clinical observation) OR Lin Chuan Shi Yan (clinical trial) OR Lian Chuang Yan Jiu (clinical research) OR Qian Zhan Xing (prospective) OR Dui Zhao (control) OR Sui Ji (random) Duo Zhong Xin (multiple centres) OR Bing Li Bao Gao (case report).

#4: #1 AND #2 AND #3.
